# Differential effects of developmental thermal plasticity across three generations of guppies (*Poecilia reticulata*): canalization and anticipatory matching

**DOI:** 10.1038/s41598-017-03300-z

**Published:** 2017-06-28

**Authors:** Amélie Le Roy, Isabella Loughland, Frank Seebacher

**Affiliations:** School of Life and Environmental Sciences A08, University of Sydney, NSW 2006 Camperdown, Australia

## Abstract

Developmental plasticity can match offspring phenotypes to environmental conditions experienced by parents. Such epigenetic modifications are advantageous when parental conditions anticipate offspring environments. Here we show firstly, that developmental plasticity manifests differently in males and females. Secondly, that under stable conditions, phenotypic responses (metabolism and locomotion) accumulate across several generations. Metabolic scope in males was greater at warmer test temperatures (26–36 °C) in offspring bred at warm temperatures (29–30 °C) compared to those bred at cooler temperatures (22–23 °C), lending support to the predictive adaptive hypothesis. However, this transgenerational matching was not established until the second (F2) generation. For other responses, e.g. swimming performance in females, phenotypes of offspring bred in different thermal environments were different in the first (F1) generation, but became more similar across three generations, implying canalization. Thirdly, when environments changed across generations, the grandparental environment affected offspring phenotypes. In females, the mode of the swimming thermal performance curve shifted to coincide with the grandparental rather than the parental or offspring developmental environments, and this lag in response may represent a cost of plasticity. These findings show that the effects of developmental plasticity differ between traits, and may be modulated by the different life histories of males and females.

## Introduction

Environmental conditions experienced by parents and during early embryonic development can modify offspring phenotypes^1–3^. Such developmental plasticity across generations (transgenerational effects) acts much quicker than adaptation by natural selection. In the context we use the term here, developmental plasticity denotes a change in the environmental sensitivity of a physiological rate in response to the environment experienced by previous generations or during early embryogenesis. Developmental plasticity is thought to be advantageous when parental environments predict those of their offspring so that offspring phenotypes are matched to the conditions experienced later in life^4–6^. Rather than modifying nucleotide sequences on DNA, epigenetic mechanisms that cause developmental plasticity alter access by transcriptional regulators to DNA and thereby alter gene expression programs^7^. DNA methylation patterns, for example, are established in the primordial germ cells and again in the pre-implantation embryos^8, 9^, and the methylation code is more or less stable beyond these very early developmental stages^3^. Other mechanisms include modifications of histones^10^, micro RNAs^11^, and maternal effects^12^. Epigenetic modifications can influence population responses to environmental change, including climate change^3, 13^, and modulate selection via genetic accommodation and assimilation^14–16^. Environmental drivers that can induce developmental plasticity include parental diet^17^, and the thermal environment^18^.

Transgenerational effects may be transmitted across several generations, and grandparental diet or behavioural conditioning, for example, can still affect phenotypes^19–21^. It has been suggested that rather than acting as a digital (on-off) mechanism, the phenotypic effects of epigenetic modifications in response to the environment occur gradually over several generations^22^. If that were the case, the potential benefits of developmental plasticity would be cumulative across several generations rather than just between parents and offspring, if the environment remained relatively stable. On the other hand, the costs of developmental plasticity could be exacerbated in variable environments. The cost of plasticity lies principally in a mismatch between phenotype and environment, when later offspring environmental conditions are different from those experienced by parents and during the early embryonic stages^6, 17, 23^. The greater the time lag between the environmental stimulus and the epigenetically-induced phenotypic response, the greater the potential for a mismatch. Our aim was to determine whether transgenerational effects in response to an environmental change accumulate across several generations. We tested the hypotheses that (a) thermal performance of offspring is matched to conditions experienced by parents and during early development so that offspring perform better in the matched relative to the mismatched environmental conditions. (b) that phenotypic responses accumulate across several generations to improve the match of performance optima to different but stable environmental conditions; (c) if the environment changes between grandparental and parental generations, the grandparental environment will influence phenotypes and attenuate phenotypic matching to environmental temperatures.

## Materials and Methods

### Study animals

**Figure 1:**
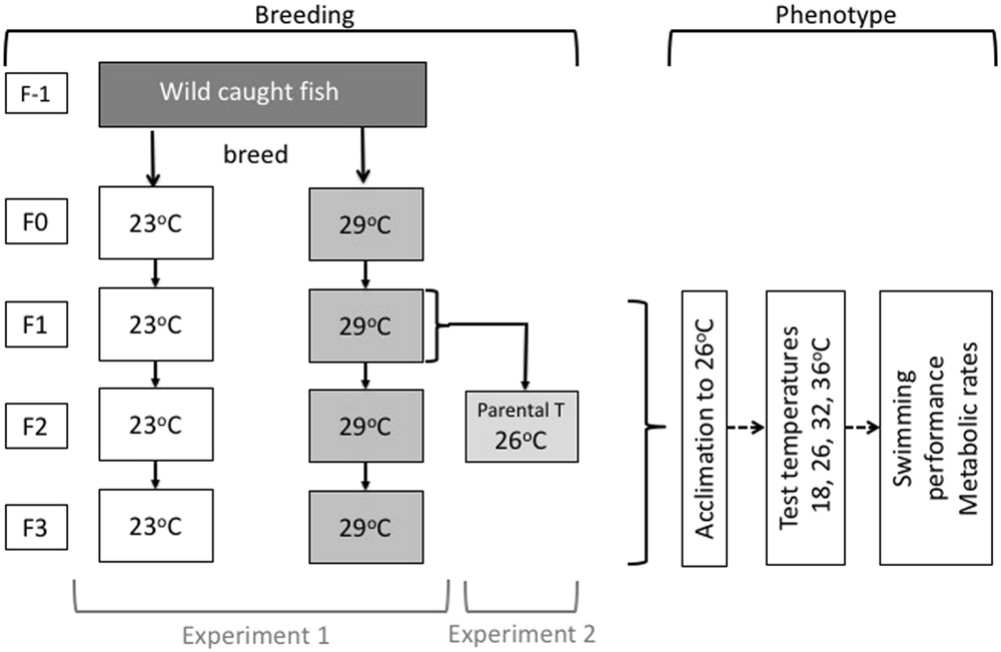
Schematic outline of the experiments. We obtained guppies (*Poecilia reticulata*) from the wild (generation F-1) and bred them in captivity. We used first generation offspring from the wild population as our experimental parental (F0) generation. We placed F0 fish as juveniles into two temperature treatments (22–23 °C [23 °C] and 29–30 °C [29 °C]). For Experiment 1, which aimed to test for cumulative thermal effects of developmental temperatures across generations, we bred F0 for a further three generations (F1 to F3) under constant conditions (23 and 29 °C). To test whether there are grandparental effects across different environments (Experiment 2), we raised and bred F1 fish from both 23 and 29 °C treatments (grandparental temperatures) at 26 °C (F2, parental temperatures), so that any differences in their offspring (F3) must have originated in the grandparental generation. All experimental fish were acclimated to 26 °C for three weeks to eliminate any effects of reversible acclimation before measuring phenotypic responses (swimming performance and metabolic rates) at the different test temperatures (18, 26, 32, 36 °C).

All procedures were performed with the approval from the University of Sydney Animal Ethics Committee (approval number L04/1–2013/3/5907) and we confirm that all methods were performed in accordance with the relevant guidelines and regulations. Guppies (*Poecilia reticulata*) are an ideal model species for this study, because they are fast breeding and offspring develop quickly; under our experimental conditions guppies reached sexual maturity within 2–3 month of age and reproduced within 3–4 months of age. Guppies were obtained from a wild population in the Northern Territory, Australia (12°25´S, 130°50´E). Fish were kept in plastic tanks (645 × 423 × 276 mm) with a density of 1–2 fish per litre at 25–26 °C with a 12 h dark: 12 h light cycle. Fish were fed twice per day with fish flakes (Wardley Tropical Fish Flakes, The Hartz Mountain Corporation, Secaucus, NJ, USA). There was an air filter (Biochemical sponge filter, Age of Aquariums, Australia) connected to an air pump (AC-9908; Resun, China) in each tank. We bred wild fish under these conditions and used their offspring as the parental fish in the experiments (Fig. 1).

### Experimental design: Experiment 1

In this experiment, we tested whether phenotypes are matched to environmental conditions, and phenotypic changes accumulate over several generations in response to different stable thermal conditions (Fig. 1). To produce our parental (F0) generation, we placed randomly selected juvenile (1–2 weeks old) fish bred from the original wild stock into either a 22–23 °C treatment (23 °C developmental temperature) or a 29–30 °C treatment (29 °C development treatment); these temperatures resemble relatively cool and warm temperatures, respectively, in tropical Australian habitats occupied by guppies. Within each treatment, fish were dispersed across 6–8 tanks (370 × 250 × 190 mm; 5–7 fish of mixed sex into each of tank). We used the offspring from the F0 fish either for experiments (F1), or to breed the next generation (F2) under the same conditions as described above. Fish were randomly allocated to the different treatments and we ensured that each treatment received fish from each of the parental tanks. We removed fish intended for experiments from the breeding tanks before they reached sexual maturity, so that we used only virgin animals in experiments to avoid confounding effects of pregnancy (see also below). This process was repeated for the F2 fish to either produce fish for experiments or to breed the F3 generation. We measured phenotypic responses (sustained locomotor performance [U_crit_], and resting and maximal metabolic rates [see below]) in 8–9 males and eight females in each developmental treatment and generation, with a total of 102 fish (23 °C developmental temperature: females, length 0.021 ± 0.00093 m, mass 0.23 ± 0.033 g; males, length 0.018 ± 0.00031 m, mass 0.11 ± 0.0066 g. 29 °C developmental temperature: females, length 0.015 ± 0.00056 m, mass 0.070 ± 0.012 g; males: 0.015 ± 0.00032 m, mass 0.072 ± 0.0047 g). The sample sizes here and below were based on previous research (e.g. ref. 24) where they were sufficient to detect differences in thermal plasticity between treatment groups.

Before measuring phenotypic responses, we acclimated fish from both developmental temperatures for three weeks to a common garden temperature (26 °C) to eliminate potential effects of reversible thermal acclimation, which could mask the effects of developmental plasticity per se^24, 25^. We placed the immature fish selected for experiments into same-sex tanks for the common-garden treatments, and fish matured during the three-week acclimation period.

We measured U_crit_ and metabolic rates across a range of acute test temperatures (18, 26, 32, and 36 °C). We chose these test temperatures based on preliminary trials measuring U_crit_ in fish not elsewhere used in the experiment. Our aim was to choose acute test temperatures that encompassed the developmental temperatures, and fell on either side of the temperature at which maximum performance occurred but without damaging the fish. In the event, swimming performance declined at 36 °C in all experimental groups, but metabolic rates did not. Nonetheless, we decided not to increase test temperatures to avoid harming the fish.

### Experimental design: Experiment 2

Here we aimed to test whether the grandparental environment influenced phenotypes when the environment changed between the grandparental and parental generations. We bred fish at 22–23 °C and at 29–30 °C to the F1 generation as described above. We then raised and bred juvenile F1 at 26 °C to the F2 generation (Fig. 1). The F2 generation therefore was derived from different grandparental temperatures (23 °C and 29 °C grandparental treatments) but the same parental temperature (26 °C), which allowed us to detect any grandparental effects on F3 offspring phenotypes. We measured phenotypic responses (sustained locomotor performance [U_crit_], and resting and maximal metabolic rates [see below]) in eight males and eight females from each grandparental temperature treatment (23 °C grandparental temperature: females, length 0.015 ± 0.00098 m, mass 0.086 ± 0.019 g; males, length 0.015 ± 0.00053 m, mass 0.074 ± 0.010 g. 29 °C developmental temperature: females, length 0.017 ± 0.00022 m, mass 0.074 ± 0.0042 g; males: 0.017 ± 0.00044 m, mass 0.072 ± 0.0023 g).

### Swimming performance

We measured swimming performance to characterise phenotypic responses, because it integrates several underlying physiological systems, and it is closely related to fitness by increasing success in predator escape, prey capture, and increasing reproductive success^26–29^. Critical sustained swimming speed (U_crit_) was measured according to published protocols^30, 31^. U_crit_ was measured in a Blazka-style swimming flume consisting of a cylindrical clear Perspex flume (150 mm length and 38 mm diameter). The flume was fitted tightly over the intake end of a submersible pump (12 V DC, iL500, Rule, Hertfordshire, UK). A bundle of hollow straws at the inlet end of flume helped maintain laminar flow. The flume and pump were submerged in a plastic tank (645 × 423 × 276 mm). We controlled water flow speed by changing the voltage input into the pump with a variable DC power source (NP9615; Manson Engineering Industrial, Hong Kong, China). The water flow in each flume was measured in real-time by a flow meter (DigiFlow 6710 M, Savant Electronics, Taichung, Taiwan) connected to the outlet of each pump. Fish swam at an initial flow rate of 0.06 m s^−1^ for 20 min followed by an increase in flow speed by 0.02 m s^−1^ every 5 min until the fish could no longer hold their position in the water column. When fish fell back onto the grid, the flow was stopped for 5–10 seconds before restarting and increasing the speed to the previous setting again. We terminated the trial when fish stopped swimming for the second time. Fish were rested for at least 24 h between swimming trials. We report U_crit_ as body length per second (BL s^−1^).

### Metabolic rate

Metabolic scope, that is the differences between resting and maximal metabolic rates, is ecologically important because it represents the energy (ATP) available for activity^32–34^. Resting metabolic rates represent the energetic costs to maintain membrane potential, protein synthesis and other processes occurring while the animal is at rest, while maximal metabolic rates reflect the maximal mitochondrial and cardiovascular capacities^33, 35^. We measured resting rates of oxygen consumption by placing individual fish inside respirometers consisting of cylindrical clear plastic tubes (15 mm diameter and 100 mm length, 27 ml volume) while a peristaltic pump (i150, iPumps, Tewkesbury, UK) circulated water through the respirometers. Resting metabolic rate was determined by measuring oxygen concentrations inside the respirometers with a sensor spot (PSt3, PreSens, Regensburg, Germany) attached to the inside of respirometers. Fiber optic cables connected to an oxygen meter (Witrox, PreSens, Regensburg, Germany) were used to monitor sensor spots. Respirometers were placed inside temperature controlled water baths, and fish were left undisturbed inside respirometers for 2 h before measuring resting oxygen consumption rates^36^. After 2 h, the respirometers were sealed by remotely switching off the pump to stop the water flow without disturbing the fish. The dissolved oxygen concentrations inside respirometers were recorded for 15–20 minutes, and the slope of oxygen depletion was used to calculate resting metabolic rates.

We measured maximum rates of oxygen consumption in each fish after measurements of resting oxygen consumption and according to a published protocol^37^. We placed individual fish in a glass cylindrical respirometer (120 ml volume) situated on a magnetic stirring plate. As above, oxygen consumption was measured by a sensor spot (PSt3, PreSens, Regensburg, Germany) attached to the inside of the glass respirometer and monitored by fiber optic cables connected to an oxygen meter (FIBOX 3, PreSens, Regensburg, Germany). A magnetic stir bar at the bottom of the respirometer created water flow, and a plastic mesh separated fish from the magnetic stir bar. A plastic column was suspended from the lid at the centre of the respirometer to help reduce turbulence. Fish were placed in the respirometer and we controlled the flow speed by adjusting the setting of the stirring plate to change the speed of the stirbar. The flow speed was increased until fish could not hold their position in the water column, after which we decreased the speed until the fish could keep their position in the water column and swim steadily. We defined this swimming speed as near maximum swimming speed. Fish swam at maximum swimming speed for approximately 10 min, and we used the slope of the decrease in oxygen concentration to calculate maximum oxygen consumption.

We measured the oxygen concentration of an empty chamber during all trials to check for other possible sources of oxygen consumption and, when necessary, we subtracted oxygen consumption of the empty chamber from the fish data. All respirometers were dried after use and regularly cleaned so that confounding effects were minimal. Metabolic scope was calculated as the difference between maximum and resting metabolic rates. We report results for metabolic scope in the main text because it is functionally most relevant, and we report resting and maximal rates of oxygen consumption in Supplementary material because these are the measurements showing how metabolic scope values were derived.

### Statistical analyses

We analysed all data with permutational analyses in the package lmPerm in R^38, 39^. We chose permutational methods because they are free of assumptions about underlying distributions, and use the data per se for statistical inference, which makes this type of analysis superior to frequentist approaches particularly when sample sizes are small (relative to the total population)^40, 41^. Briefly, permutational analysis randomizes the data set while retaining the data structure (treatment groups and numbers of samples within treatments) to generate all possible permutations of the values obtained in the experiment. Each randomized dataset is then compared to the actual dataset to assess whether the treatment effects are the same or greater in the randomized dataset compared to the actual dataset. Probabilities are calculated as the number of random data sets that have the same or greater effect as the measured data set divided by the total number of permutations. The null hypothesis is that the treatment effects in the experimental dataset are no greater than in the randomized datasets^40^. In other words, if the null-hypothesis is true, the sums of squares observed in the data are the same for all permutations of the dataset^42^ Hence, permutational analyses do not make assumptions about underlying distributions and therefore do not have a test statistic such as a t or F value. lmPerm uses type III sums of squares and implements analysis of variance models, but calculates permutation probabilities.

In the analysis for Experiment 1, we used generation (F1, F2, F3), developmental temperature (23 or 29 °C), acute test temperature (18, 26, 32, 36 °C) and sex (male and female) as fixed factors. In Experiment 2, we used grandparental developmental temperature (23 or 29 °C), sex, and acute test temperatures as fixed factors. We used standard length as covariate for analyses of swimming performance, and mass as covariate for analyses of oxygen consumption rates. Additionally, we used fish id as a random factor to account for repeated measures of the same individual at different test temperatures, using a random intercept model. When sex was significant, we performed follow-up analyses for males and females separately. We further analysed significant interactions by comparing marginal means with post-hoc permutational analyses. In analyses of U_crit_ we added test temperature as a quadratic term (Test + Test^2). Unlike parametric ANOVA models in R, lmPerm collects together the appropriate terms in response surface models and produces a correct ANOVA, so that ‘Test’ in our results Tables for U_crit_ represents the quadratic term. We did not use a quadratic term for analyses of metabolic rates, because metabolic responses to temperature were approximately linear.

Additionally, we analysed individual thermal performance curves of U_crit_ to test whether the mode (i.e. the temperature at which maximum performance occurs) of performance curves shifted as a result of different developmental temperatures, which would indicate environmental matching or mismatching across generations. We fitted quadratic equations^43^ to thermal performance curves of individual fish to determine the mode. We also determined performance breadth as the temperature range within which fish performed within 80% of maximal as described in^31^. We analysed mode and breadth of performance curves with permutational analyses of variance. The independent factors were the same as above except for test temperature, which was instead used to calculate performance curves. Note that the mode and breadth of the performance curve were calculated from four measurements of swimming performance in each fish. Hence, there may be an error associated with the estimates, although we ensured that the same person conducted swimming trials using the same equipment for each measurement.

## Results

### Experiment 1: phenotypic changes across generations within environments

#### Swimming performance

**Figure 2:**
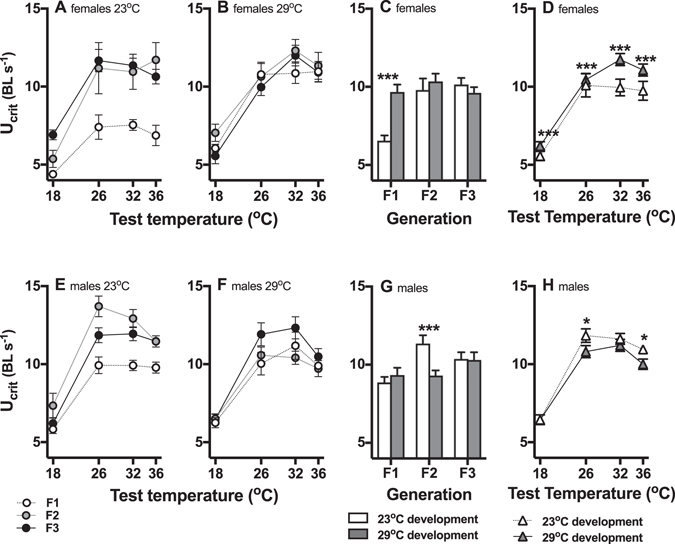
Swimming performance (U_crit_) curves across generations in different stable environments (Experiment 1). The top row of panels shows means at different test temperautres (±s.e.; **A**,**B**) and interaction plots of marginal means (±s.e.) for significant interactions (**C**, **D**) for females, and the bottom row shows means (**E**,**F**) and interaction plots (**G**,**H**) for males. There were significant differences in mean U_crit_ between females (**A**,**B**) and males (**E**,**F**). In females, U_crit_ was determined by a three way interaction between generations (F1 = open circles, F2 = grey circles, F3 = black circles), developmental conditions (**A**: 23 °C development, B: 29 °C development), and test temperature. The interaction plot (**E**) shows that U_crit_ was significantly different between developmental treatments in the F1 generation but not in subsequent generations (open bars = 23 °C developmental temperature, filled bars = 29 °C developmental temperature). Additionally, fish developed at 29 °C had greater U_crit_ than those developed at 23 °C at all test temperatures (**D**). In males, mean U_crit_ (**E**,**F**) was determined by interactions between generation and developmental temperatures, and between developmental temperature and test temperatures. In males, U_crit_ differed between developmental treatments in the F2 generation but not in the F1 or F3 generations (**G**). U_crit_ was significantly higher in fish from the 23 °C developmental temperature treatment at 26 °C and 36 °C test temperatures (**H**; open triangles = 23 °C developmental temperature, filled triangles = 29 °C developmental temperature). N = 8–9 fish per treatment group; * p < 0.05, ** p < 0.01, *** p < 0.001.

Males had overall higher sustained swimming performance (U_crit_) than females (main effect of sex, Table 1, Fig. 2A-B, E-F), and there were three-way interaction between generation, developmental temperature, test temperature, and sex, generation and developmental temperature (Table 1).

**Table 1:** Permutational analyses of swimming performance and metabolic scope from males and females in Experiment 1.

	d.f.	Ucrit	Scope
Gen	2	<0.001	<0.001
Dev	1	<0.001	0.76
Sex	1	<0.001	<0.001
Test	1(2)	<0.001	0.97
Gen*Dev	2	<0.001	<0.001
Gen*Sex	2	0.67	0.022
Gen*Test	2	0.17	0.060
Dev*Sex	1	0.88	0.98
Dev*Test	1	0.020	<0.001
Sex*Test	1	0.11	0.43
Gen*Dev*Sex	2	0.20	0.11
Gen*Dev*Test	2	0.020	<0.001
Gen*Sex*Test	2	0.019	0.88
Dev*Sex*Test	1	0.36	0.028
Gen*Dev*Sex*Test	2	0.097	0.086
Residual	381		

Permutational probabilities are shown for analyses of critical sustained swimming speed (U_crit_) and metabolic scope (Scope). The fixed factors were generation (Gen; F1-F3), developmental temperature (Dev; 23 °C and 29 °C), sex (male and female), and test temperature (Test; 18, 26, 32, and 36 °C). Degrees of freedom (d.f.) are shown; note that Test for U_crit_ has one extra d.f. because we used a quadratic term in the model. Please see main text for details of analyses.

In separate analyses of each sex, U_crit_ of females was determined by a three-way interaction between generation, developmental temperature, and test temperature (Table 2; Fig. 2A-B). Analysis of marginal means showed that U_crit_ of fish from the 23 °C developmental temperature was lower than that of fish from the 29 °C developmental temperature in the F1 generation (post-hoc p < 0.001), but not in the F2 or F3 generations (post-hoc both p > 0.9; Fig. 2C). However, across all generations U_crit_ was higher in fish developed at 29 °C compared to those developed at 23 °C at all test temperatures (post-hoc all p < 0.001; Fig. 2D).

**Table 2:** Permutational analyses of swimming performance and metabolic scope conducted separately for males and females in Experiment 1.

	d.f.	Females		Males	
		U_crit_	Scope	Ucrit	Scope
Gen	2	0.045	<0.001	<0.001	<0.001
Dev	1	<0.001	0.020	<0.001	<0.001
Test	1(2)	<0.001	0.75	<0.001	0.92
Gen*Dev	2	0.0060	<0.001	<0.001	<0.001
Gen*Test	2	0.12	0.43	0.23	0.15
Dev*Test	1	0.11	<0.001	0.0090	0.66
Gen*Dev*Test	2	<0.001	0.26	0.98	<0.001
Residuals	197				

Permutational probabilities are shown for analyses of critical sustained swimming speed (U_crit_) and metabolic scope (Scope). The fixed factors were generation (Gen; F1-F3), developmental temperature (Dev; 23 °C and 29 °C), and test temperature (Test; 18, 26, 32, and 36 °C). Degrees of freedom (d.f.) are shown; note that Test for U_crit_ has one extra d.f. because we used a quadratic term in the model. Please see main text for details of analyses.

U_crit_ of males was determined by an interaction between generation and developmental temperature (Table 2; Fig. 2E, F), and U_crit_ was significantly higher in F2 fish from the 23 °C treatment compared to the 29 °C treatment (post-hoc p < 0.0001; Fig. 2G), but there were no differences between treatments in the F1 or F3 generations (post-hoc both p > 0.3; Fig. 2G). Additionally, a significant interaction between developmental and test temperatures (Table 2) showed that U_crit_ of males from the 23 °C development treatment was significantly higher at 26 °C and 36 °C test temperatures (both post hoc p < 0.04; 18 and 32 °C, p > 0.5) compared to fish from the 29 °C treatment (Fig. 2H); this pattern was the reverse to that observed in females (cf. Fig. 2D).

The performance curves of U_crit_ were similar between treatments, and there were no significant differences between generations, developmental temperatures, or sexes in the mode (mean ± s.e. = 30.88 ± 0.33; all p > 0.15), or in performance breadth (mean ± s.e. = 17.16 ± 0.47; all p > 0.12). In summary, U_crit_ was determined by developmental temperatures, but that effect changed between generations and differed between sexes, although the thermal sensitivity (performance curves) was not affected by any experimental factor.

#### Metabolism

**Figure 3:**
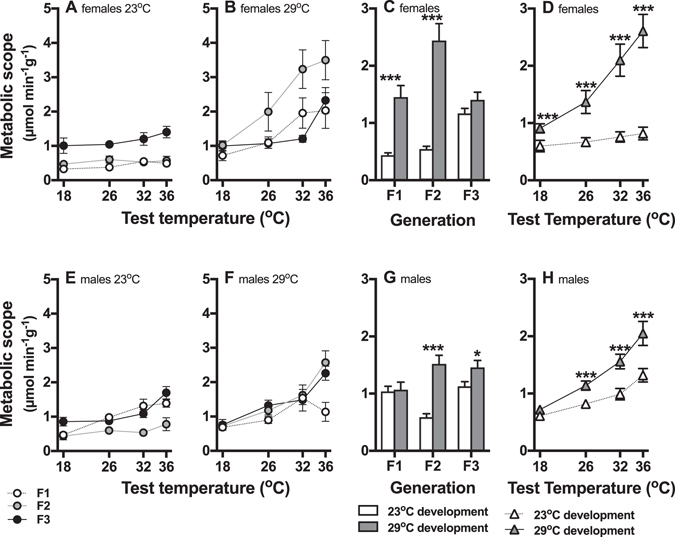
Metabolic scope of fish across generations in different stable environments (Experiment 1). The top row of panels shows means at different test temperatures (±s.e.; **A**,**B**) and interaction plots of marginal means (±s.e.) for significant interactions (**C**,**D**) for females, and the bottom row shows means (**E**,**F**) and interaction plots (**G**,**H**) of males. In females, metabolic scope was determined by interactions between generations (F1 = open circles, F2 = grey circles, F3 = black circles) and developmental conditions (**A**: 23 °C development, **B**: 29 °C development), and between developmental temperature and test temperature. The interaction plots show that metabolic scope differed significantly between developmental treatments in the F1 and F2 generations but not in F3 generation (**C**; open bars = 23 °C developmental temperature, filled bars = 29 °C developmental temperature), and that fish developed at 29 °C (**D**; filled triangles) had greater metabolic scope than those developed at 23 °C (open triangles). In males, metabolic scope was determined by a three-way interaction between generation, developmental temperature (**E**: 23 °C development, F: 29 °C development), and test temperature. In the opposite patterns to females, in males metabolic scope differed between developmental treatments in the F2 and F3 generations but not in the F1 generation (**G**). Metabolic scope was significantly higher in fish from the 29 °C developmental temperature treatment at 26 °C, 32 °C and 36 °C test temperatures (H; open triangles = 23 °C developmental temperature, filled triangles = 29 °C developmental temperature). N = 8–9 fish per treatment group; * p < 0.05, ** p < 0.01, *** p < 0.001.

Similar to U_crit_, responses of resting and maximal rates of oxygen consumption (Supplementary Fig. 1), and metabolic scope (Fig. 3) differed between sexes (main effects, and interactions between sex and generation, developmental temperature and test temperature (Table 2, Supplementary Table 1) so that we analysed data from males and females separately. Resting and maximal metabolic rates of both females and males were determined by three-way interactions between generation, developmental temperature, and test temperature (Supplementary Fig. 1, Supplementary Table 1).

In females, metabolic scope was determined by interactions between generations and developmental temperature (Table 2; Fig. 3A,B), and between developmental temperature and test temperature (Table 2, Fig. 3A,B). The interaction plots show that metabolic scope differed significantly between developmental treatments in the F1 and F2 generations (both post-hoc p < 0.001) but not in F3 generation (post-hoc p = 0.35; Fig. 3C), and that fish developed at 29 °C had greater metabolic scope than those developed at 23 °C (Fig. 3D).

Metabolic scope of males was determined by a three-way interaction between generation, developmental temperature, and test temperature (p < 0.0001; Table 2, Fig. 3E,F). Unlike females, in males metabolic scope differed between developmental treatments in the F2 (post-hoc p < 0.001) and F3 (post-hoc p = 0.020) generations but not in the F1 generation (post-hoc p = 0.92; Fig. 3G). Metabolic scope was significantly higher in fish from the 29 °C developmental temperature treatment at 26 °C, 32 °C and 36 °C test temperatures (all post-hoc p < 0.001; Fig. 3H).

### Experiment 2: grandparental effects across environments

#### Swimming performance

**Figure 4:**
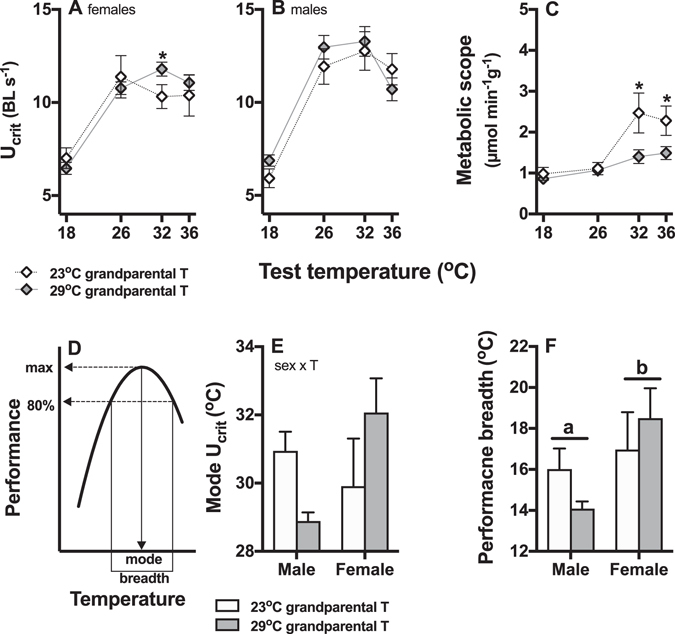
Swimming performance (U_crit_) and metabolic scope of F2 fish derived from grandparents that experienced different environments (Experiment 2). There were significant differences between females (**A**) and males (**B**) in U_crit_, but not in metabolic scope (**C**). In females, U_crit_ of fish from grandparents bred at 23 °C (open diamonds) was lower at 32 °C test temperature (indicated by asterisk) than that of fish from grandparents bred at 29 °C (black diamonds; grandparental x test temperature interaction). In males, there was a main effect of test temperature, and U_crit_ was lower in fish from grandparents bred at 23 °C (main effect of grandparental temperature). Metabolic scope (**C**) did not differ between sexes, but metabolic scope of fish from grandparents bred at 23 °C was significantly higher (indicated by asterisks) at 32 and 36 °C test temperature (interaction between grandparental and test temperature). In the analysis of individual thermal performance curves (**D**), the mode denotes the temperature at which the maximum performance occurs, and the breadth identifies the temperature range within which performance is greater than 80% of maximum. Grandparental temperature had different effects on the mode of the performance curves between males and females (**E**), grandparental temperature x sex interaction) and females from 29 °C bred grandparents had a higher mode than those from 23 °C grandparents, but the reverse was the case for males. Males had lower performance breadth (indicated by different letters) than females (**F**), but there was no effect of grandparental temperature. Means ± s.e. are shown and N = 7–9 fish per treatment group.

U_crit_ was determined by a three-way interaction between grandparental temperature, sex, and test temperature (Table 3), and we therefore analysed data from different sexes separately (Fig. 4A,B). In females derived from 29 °C grandparental treatments, U_crit_ was higher at the higher temperatures (grandparental temperature x test temperature interaction, Table 4; significant difference at 32 °C, post-hoc p = 0.03; Fig. 4A). In males, U_crit_ changed with test temperature (main effect of test temperature, Table 4), and it was overall higher in fish derived from 29 °C grandparental treatments (main effect of grandparental temperature; Table 4; Fig. 4B).

**Table 3:** Permutational analyses of swimming performance and metabolic scope for males and females in Experiment 2.

	d.f.	U_crit_	Scope
Gran	1	<0.001	0.0080
Sex	1	<0.001	0.062
Test	1(2)	<0.001	<0.001
Gran*Sex	1	0.12	0.12
Gran*Test	1	0.97	0.034
Sex*Test	1	0.58	0.94
Gran*Sex*Test	1	0.019	0.82
Residual	114		

Permutational probabilities are shown for analyses of critical sustained swimming speed (U_crit_) and metabolic scope (Scope). The fixed factors were grandparental temperature (Gran; 23 °C and 29 °C), sex (male and female), and test temperature (Test; 18, 26, 32, and 36 °C). Degrees of freedom (d.f.) are shown; note that Test for U_crit_ has one extra d.f. because we used a quadratic term in the model. Please see main text for details of analyses.

**Table 4:** Permutational analyses of swimming performance and metabolic scope conducted separately for males and females in Experiment 2.

	d.f.	Females	Males
		U_crit_	U_crit_
Gran	1	0.35	<0.0054
Test	2	<0.001	<0.001
Gran*Test	1	0.010	0.28
Residuals	57		

Permutational probabilities are shown for analyses of critical sustained swimming speed (U_crit_). The independent factors were grandparental temperature (Gran; 23 °C and 29 °C), and test temperature (Test; 18, 26, 32 and 36 °C). Degrees of freedom (d.f.) are shown. Please see main text for details of analyses.

The mode of the U_crit_ thermal performances curve (Fig. 4D) was higher in males from the 23 °C grandparental group than in those from the 29 °C grandparental group, and the reverse was the case for females (Fig. 4E, grandparental temperature x sex interaction p < 0.02). Females had greater performance breadth than males regardless of grandparental temperatures (main effect of sex p < 0.05, grandparental temperature and interactions p > 0.15; Fig. 4F).

#### Metabolism

Resting oxygen consumption was determined by a three-way interaction between sex, grandparental temperature, and test temperature (Supplementary Table 3; Supplementary Fig. 3). In males, resting oxygen consumption changed with test temperature only (Supplementary Table 4), while in females it was determined by an interaction between grandparental and test temperatures (Supplementary Table 4, Supplementary Fig. 3A, B). Maximal rates of oxygen consumption did not differ between sexes, but were determined by the interaction between grandparental and test temperatures (Supplementary Table 3; Supplementary Fig. 3C,D).

There was no significant difference between sexes in metabolic scope (Table 3), but metabolic scope was determined by an interaction between grandparental temperature and test temperature (Table 3; Fig. 4C). Compared to fish from the 29 °C grandparental treatment, metabolic scope was higher at 32 °C (post-hoc p = 0.022) and 36 °C (post-hoc p = 0.028) test temperatures in fish derived from grandparents bred at 23 °C (Fig. 4C).

## Discussion

We have shown that under stable environmental conditions, phenotypic changes accumulate over three generations. Our data provide some support for the hypothesis that parental environments match offspring phenotypes to their environment. Our results are consistent with the predictive adaptive hypothesis in metabolic scope of males, which was higher at high test temperatures when fish were bred at high temperatures. However, cold-developed fish did not perform better at low test temperatures so that the support for the predictive adaptive hypothesis is limited. Interestingly, however, these differences between developmental environments were apparent only in the F2 generation, which indicates that the effects of developmental plasticity accumulate over more than one generation. Female metabolic scope was higher at all test temperatures in fish bred at the warm temperature, and differences between developmental treatments disappeared by the F3 generation. These results are reminiscent of canalization where phenotypic variation remains low despite genetic and environmental variation^44, 45^. The greater performance of females bred at 29 °C across all test temperatures resembles a “warmer-is-better” response^46^, which could be beneficial for a tropical species like guppies.

Differences in developmental conditions could be viewed as a perturbation of phenotypes (e. g. U_crit_ of females), which leads to differences in the F1 or F2 generations. However, optimal phenotypes are re-established by the third generation regardless of differences in environmental conditions. For example, the relatively low swimming performance in the F1 generation at 23 °C developmental temperature may reflect a thermodynamic depression of physiological mechanisms underlying locomotion^47–49^. The fish respond to this depression over the next two generations so that physiological function and swimming performance increase to the same levels as that seen in fish that developed at 29 °C. Similar responses to depressing thermodynamic effects occur during reversible thermal acclimation within individuals and may involve increases in mitochondrial density and complexity and therefore increased flux through mitochondrial pathways^50^, changes in membrane composition^51, 52^, modifications of muscle phenotypes^53–55^, and cardiovascular adjustments^56^. Our results show that within the same species and population, developmental plasticity can manifest both as anticipatory (grand) parental matching of phenotypes^5, 6^, and canalization which buffers or ‘protects’ phenotypes from potentially negative environmentally-induced changes^57, 58^.

Transmission of environmentally-induced phenotypes across generations can be advantageous by buffering phenotypes from the effects of stochastically fluctuating environments, or when ancestral parental or grandparental environments predict current environments^59^. However, such phenotypic memory can be detrimental when mean environmental conditions change across generations^23, 59, 60^. We have shown that grandparental environments influence offspring phenotypes even when there is a mismatch between these generations. Interestingly, the grandparental environment affected both the mode of U_crit_ thermal performance curves, and their breadth. In female offspring, the shift in the mode of the performance curve was proportional to the grandparental environment, that is, it was higher in offspring with grandparents from warm temperatures. This lag in effect of the grandparental environment by one generation can represent a cost of developmental plasticity when environments change between generations as in our experiments. Similarly, rapid environmental changes such as those resulting from natural phenomena such as El Nino^61^ or from human-induced climate change^62^, could cause a mismatch between a phenotype and its environment if the phenotypic response is established across several generations.

The difference between the sexes we observed consistently is interesting, because it shows that males and females not only differ in absolute trait values but also in the degree to which those trait values are influenced by previous generations. Male and female guppies have very different life history trajectories and respond differently to ecological processes such as predation^63^. The lifespan of males is shorter than that of females^64^, so that males may also experience a narrower range of lifetime environmental conditions, which may make anticipatory (grand)parental effects more advantageous. Additionally, females store sperm which effectively lengthens the reproductive age of males beyond the death of the individual^64^. It would be very interesting to investigate whether sperm storage alters epigenetic effects on offspring because the delay in fertilization may increase the risk of an environmental mismatch.

The coercive mating system of guppies also means that selection for performance traits differs between sexes, and reproductive behaviour in guppies can influence physiological responses^65^. The perpetual escape from males may lead to greater locomotor activity and physical training effects as well as increased sex-specific selection for traits underlying locomotion^65^. The costs of sexual conflict may be reduced by plastic responses that increase locomotor efficiency rather than absolute swimming speed^65^. Sex-specific demands for locomotor performance may therefore at least partly explain the differences we observed between males and females. Social interactions and behaviours such as aggression may be mediated by (neuro)endocrine factors, such as corticosteroids, that also influence metabolism^34, 66–68^. The strongest indication of anticipatory parental matching of offspring phenotypes we observed was in metabolic scope of males. Reproductive success in male guppies may rely on success in intra-specific conflict and competition with other males^69^, as well as success in coercive mating^70^. Metabolic scope may enable success in these social interactions. However, in different thermal environments there may be strong selection to maximise the efficacy of neuroendocrine pathways that influence behavioural phenotypes, and thereby also metabolism secondarily.

DNA methylation codes are laid down during gametogenesis and during very early embryogenesis^8^. There are pronounced differences between males and females in gamete development and their epigenetic state^71^. In starlings, for example, rainfall patterns during early development influenced DNA methylation patterns of glucocorticoid receptor genes in a sex-specific manner, and thereby affected fitness of males and females differentially^72^. Hence, the environment can impact epigenetic profiles of males and females differentially, and thereby cause sex-specific phenotypic trajectories across generations. These gender-specific responses are important experimentally, because a clear distinction has to be made between the sexes, and ecologically because environmental change can have fundamentally different effects on males and females. The differences we observed between sexes in developmental plasticity will be important to pursue further, because they may also influence populations dynamics in changing environments.

It is possible that selection played a role in obtaining our results, assuming that the phenotypic traits we measured are heritable. For example, females that are better adapted genetically to either developmental temperature may have contributed a greater proportion of offspring to the next generation. If that were the case, however, it would be expected that changes in phenotype are directional across generations within the same environment, and that fish would become increasingly specialised to their environment. We rarely observed these responses in our data. However, it should be noted that the developmental modifiers that mediate epigenetic changes, such as DNA methyltransferases (DNMT), have themselves evolved so that there always will be a genetic component in epigenetic responses^73^. In environments that fluctuate between generations, selection should favour modifiers that reduce phenotypic expression of underlying genetic variance, because responses to selection in one generation would be maladaptive in the next^44^.

Additionally, it is also possible that differences in offspring phenotypes did not arise from the action of developmental modifiers such as DNMT enzymes, but from other parental effects such as provisioning embryos with cellular organelles, or producing different sized offspring in different environments^12, 74^. The immediate offspring environment early in development may also affect phenotypes when, for example, conditions are extreme and cause physiological damage or, vice versa, favourable conditions during early development may lead to better performing adults^74, 75^. Similar to above, however, if these factors played a role in the responses of our guppies it would have been expected that there were consistent differences between fish from different developmental temperature treatments across generations, because the environments stayed constant. Hence, even though we cannot rule out an effect of selection, maternal effects, or direct environmental impacts on the embryos, these alone cannot explain our results. It is likely, therefore, that mechanisms such as DNA methylation^8, 76^, histone modifications^10^, or micro RNAs^11^ played a role in determining phenotypes. Disentangling the cause and effect underlying developmental plasticity will be important in understanding the evolution of plasticity and its responsiveness to environmental variability.

Our findings that the effects of developmental plasticity manifest differently in different traits and sexes and do not necessarily match offspring phenotypes to their environment have implications for understanding the evolution of developmental plasticity, because the benefits are not as clearly defined as stipulated by the predictive adaptive hypothesis, for example. Rather, it is possible that the evolution of developmental plasticity is trait-specific and is modified by the roles those traits play in life history.

### Data accessibility

The complete data set has been submitted as Supplementary Material.

## Electronic supplementary material

Supplementary Information

Supplementary Dataset 1
