# Supplementary material for: Differential effects of developmental thermal plasticity across three generations of guppies (Poecilia reticulata): canalization and anticipatory matching
Source: Sci Rep. 2017 Jun 28;7:4313. doi: 10.1038/s41598-017-03300-z (PMC5489511; doi:10.1038/s41598-017-03300-z)
Supplement: Supplementary file 1 — Supplementary Information [file 41598_2017_3300_MOESM1_ESM.doc]

**Differential effects of developmental thermal plasticity across three generations of guppies (*Poecilia reticulata*): canalization and anticipatory matching**

**Supplementary Material**

**Amélie Le Roy, Isabella Loughland, Frank Seebacher***

*School of Life and Environmental Sciences A08, University of Sydney, NSW 2006, Australia*

*author for correspondence: frank.seebacher@sydney.edu.au

**Supplementary results**

*Experiment 1: metabolic rates*

Responses of resting and maximal rates of oxygen consumption (Supplementary Fig. 1) differed between sexes (significant interactions between sex and generation, developmental temperatures and test temperatures, Supplementary Table 1), so that we analysed data from males and females separately. Resting and maximal metabolic rates of both females and males were determined by three-way interactions between generation, developmental temperature, and test temperature (Supplementary Table 2).

Resting and maximal rates of oxygen consumption of females were significantly higher in F1 and F2 - but not F3 - fish from the 29^o^C development treatment compared to 23^o^C (post-hoc analysis of marginal means; Supplementary Fig. 2 A, C). Additionally, resting and maximal rates were higher across all test temperatures (except at 36^o^C for resting rates) in female fish from the 29^o^C development treatment compared to 23^o^C (post-hoc all p < 0.05; Supplementary Fig. 2 B, D).

In males, resting and maximal metabolic rates were significantly higher in F2 fish from the 29^o^C development temperature treatment compared to the 23^o^C developmental temperature treatment (post-hoc p = 0.0054 and p < 0.001, respectively; Supplementary Fig. 2 E, F). Similar to females, resting and maximal rates were higher in males from the 29^o^C developmental treatment at all test temperatures except for 18^o^C compared to fish from the 23^o^C developmental temperature treatment (post-hoc all p < 0.05; Supplementary Fig. 2F, H).

*Experiment 2: metabolic rates*

Resting metabolic rates were determined by a three-way interaction between grandparental developmental temperature, test temperature (Supplementary Table 3). In females, resting metabolic rates were significantly higher at 32 and 36^o^C acute test temperature in fish derived from grandparents bred at 23^o^C, compared to fish derived from grandparents bred at 29^o^C (grandparental x test temperature interaction, Table 4, post hoc p = 0.043 and p < 0.001, respectively; Supplementary Fig. 3A). Resting metabolic rate of males were determined by test temperature only (Supplementary Table 4; Supplementary Fig. 3B).

Maximal metabolic rates did not differ between sexes (Supplementary Table 3; Supplementary Fig. 3C), but were determined by an interaction between grandparental and test temperatures (Supplementary Table 3). In fish derived from grandparents bred at 23^o^C, maximal metabolic rates were significantly higher at 32 and 36^o^C test temperature (post-hoc p = 0.017 and p = 0.043, respectively) compared to fish from 29^o^C grandparental temperatures (Supplementary Fig. 3C).

**Supplementary Tables**

**Supplementary Table 1** Statistical results of the full models from Experiment 1. Permutational probabilities are shown for analyses of resting metabolic rates (Rest) and maximal metabolic rates (Max). The independent factors were generation (Gen; F1-F3), developmental temperature (Dev; 23^o^C and 29^o^C), sex (male and female), and test temperature (Test; 18, 26, 32, and 36^o^C). Degrees of freedom (d.f.) are shown. Please see main text for details of analyses.

|  | d.f. | Rest | Max |
| --- | --- | --- | --- |
| Gen | 2 | 0.46 | <0.001 |
| Dev | 1 | 0.15 | 0.98 |
| Sex | 1 | 0.54 | <0.001 |
| Test | 1 | <0.001 | <0.001 |
| Gen*Dev | 2 | 0.12 | <0.001 |
| Gen*Sex | 2 | 0.79 | 0.039 |
| Gen*Test | 2 | 0.0080 | <0.001 |
| Dev*Sex | 1 | 0.25 | 0.11 |
| Dev*Test | 1 | <0.001 | 0.98 |
| Sex*Test | 1 | 0.036 | 0.63 |
| Gen*Dev*Sex | 2 | 0.15 | 0.030 |
| Gen*Dev*Test | 2 | 0.064 | <0.001 |
| Gen*Sex*Test | 2 | 0.043 | 0.48 |
| Dev*Sex*Test | 1 | 0.013 | 0.49 |
| Gen*Dev*Sex*Test | 2 | 0.024 | 0.19 |
| Residual | 382 |  |  |

**Supplementary Table 2** Statistical results from Experiment 1. Permutational probabilities are shown for analyses of resting metabolic rates (Rest) and maximal metabolic rates (Max). The independent factors were generation (Gen; F1-F3), developmental temperature (Dev; 23^o^C and 29^o^C), and test temperature (Test; 18, 26, 32, and 36^o^C). Degrees of freedom (d.f.) are shown. Please see main text for details of analyses.

|  |  | Females | | Males | |
| --- | --- | --- | --- | --- | --- |
|  | d.f. | Rest | Max | Rest | Max |
| Gen | 2 | 0.64 | <0.001 | 0.39 | <0.001 |
| Dev | 1 | 0.98 | 0.024 | 0.050 | <0.001 |
| Test | 1 | <0.001 | <0.001 | <0.001 | <0.001 |
| Gen*Dev | 2 | 0.53 | <0.0042 | <0.001 | <0.001 |
| Gen*Test | 2 | 0.0032 | 0.0052 | 0.42 | <0.001 |
| Dev*Test | 1 | <0.001 | 0.82 | 0.089 | 0.71 |
| Gen*Dev*Test | 2 | 0.0044 | 0.0018 | 0.0072 | <0.001 |
| Residuals | 182 |  |  |  |  |

**Supplementary Table 3** Statistical results of the full models from Experiment 2. Permutational probabilities are shown for analyses of resting metabolic rate (Rest) and maximal metabolic rate (Max). The independent factors were grandparental temperature (Gran; 23^o^C and 29^o^C), sex (male and female), and test temperature (Test; 18, 26, 32, and 36^o^C). Degrees of freedom (d.f.) are shown. Please see main text for details of analyses.

|  | d.f. | Rest | Max |
| --- | --- | --- | --- |
| Gran | 1 | 0.57 | 0.0034 |
| Sex | 1 | 0.23 | 0.67 |
| Test | 1 | <0.001 | <0.001 |
| Gran*Sex | 1 | 0.35 | 0.17 |
| Gran*Test | 1 | 0.080 | 0.0066 |
| Sex*Test | 1 | 0.054 | 0.19 |
| Gran*Sex*Test | 1 | 0.026 | 0.93 |
| Residual | 114 |  |  |

**Table 4** Statistical results from separate analyses of data from males and females in Experiment 2. Permutational probabilities are shown for analyses of resting metabolic rate (Rest). The independent factors were grandparental temperature (Gran; 23^o^C and 29^o^C), and test temperature (Test; 18, 26, 32, and 36^o^C). Degrees of freedom (d.f.) are shown. Please see main text for details of analyses.

|  |  | Females | Males |
| --- | --- | --- | --- |
|  | d.f. | Rest | Rest |
| Gran | 1 | 0.14 | 0.72 |
| Test | 1 | <0.001 | <0.001 |
| Gran*Test | 1 | 0.019 | 0.94 |
| Residuals | 57 |  |  |

**Supplementary Figures**

**Supplementary Fig. 1** Resting and maximal metabolic rates measured in Experiment 1. Resting and maximal metabolic rates of fish from the F1 (open circles), F2 (grey circles) and F3 (black circles) were significantly different between females (A-D) and males (E-H). Resting and maximal metabolic rates of both females and males were determined by three-way interactions between generation, developmental temperature, and test temperature. Means ± s.e. are shown, and N = 8-9 fish per treatment group.

**Supplementary Fig. 2** Interaction plots showing marginal means. Resting and maximal metabolic rates were determined by interactions between developmental temperatures (open triangles or bars = 23^o^C developmental temperature, closed triangles or bars = 29^o^C developmental temperature) and generations (females A and C, respectively; males E and G, respectively), and between developmental and test temperatures (females B and D, respectively; males F and H respectively). Asterisks indicate significant differences (* < 0.05; ** < 0.01, *** < 0.001) between marginal mean (± s.e.).

**Supplementary Fig. 3** Resting and maximal metabolic rates measured in Experiment 2. Resting metabolic rates differed between sexes. In females (A) resting rates were higher at 32 and 36^o^C test temperatures in fish derived from 23^o^C grandparental temperatures (open diamonds) compared to 29^o^C grandparental temperatures (black diamonds). In males (B), resting rates increased with test temperature only, and grandparental temperature had no effect. Maximal metabolic rates (C) did not differ between sexes, but were determined by an interaction between grandparental and test temperatures. Means ± s.e are shown and N = 7-9 fish per treatment group.
